# The Moderating Effect of Community-Level Deprivation on the Association between Individual Characteristics and Smoking Behavior among Chinese Adults: A Cross-Level Study

**DOI:** 10.3390/ijerph18115785

**Published:** 2021-05-27

**Authors:** Nan Chen, Chang-Gyeong Kim

**Affiliations:** 1 Major in Chinese Studies, Department of Global Business, Kosin University, Busan 49104, Korea; chennana@kosin.ac.kr; 2 Department of Chinese Studies, Pukyong National University, Busan 48513, Korea

**Keywords:** community deprivation, perceived stress, socioeconomic status, smoking intensity, multilevel analysis, China Health and Nutrition Survey (CHNS), China

## Abstract

China joined the World Health Organization (WHO) Framework Convention on Tobacco Control in 2006; however, the overall Chinese smoking rate is still high. The aim of this study is to provide new evidence for the direct effects of community-level deprivation, and the effects of interactions between community-level deprivation and individual characteristics, on smoking intensity, by using cross-sectional data from the 2015 China Health and Nutrition Survey (CHNS). The results show that there is a strong association between community-level deprivation and individual smoking intensity, and that community deprivation moderates the relationship between individual perceived stress and smoking intensity. The findings imply that adequate interventions should be conducted in the context of deprived neighborhoods, and should consider differences between levels of individual perceived stress and between sexes, especially focusing on highly stressed women who live in deprived communities.

## 1. Introduction

People’s health-related behaviors depend on the context in which they live [1]. Context is a compound formation including individual resources and the combination of circumstances. Within this perspective, influences on smoking behaviors may range from individual characteristics to the community context [2,3,4]. Given these multiple influences, a cross-level framework is proposed to examine the correlations between the microscale of people and the macroscale of contextual settings in terms of smoking behavior [5].

A community-level predictor reflects “the wider social structures that operate to constrain or enable human behaviors” [6]. Neighborhood deprivation is considered an important contextual factor in shaping individual smoking behavior. Previous studies showed that individuals who reside in deprived neighborhoods are more likely to begin smoking due to their disadvantaged neighborhood settings [7,8,9]. These neighborhood settings include socially interactive, environmental, geographical, and institutional mechanisms that may influence smoking prevalence through behaviors rooted in neighborhood-level peer interaction, substandard physical features, inadequate basic resources, and organizational entities such as the density of tobacco retailers [10,11]. The community context can affect personal smoking behavior beyond the impact of individual characteristics [12]. Some studies conducted in Western society found that the most deprived quintiles of neighborhoods had the highest densities of tobacco outlets and supply, which may be a contributing factor to tobacco use [13].

Regarding individual-level characteristics, perceived stress and individual social status were found to independently correlate with smoking behavior [14,15]. Through a cross-level approach, some studies found that smoking behaviors influenced by neighborhood deprivation differ by individual educational level, occupational social class, and gender [16,17]. Others revealed that individual perceived stress leads to unhealthy behaviors in deprived neighborhoods [18,19]. Despite this evidence, empirical studies investigating the influence of individual characteristics and environmental factors on smoking behavior remain poorly understood in Asian societies. Although some cross-level studies conducted in Asia found a relationship between neighborhood contextual effects and smoking behavior [20,21,22,23], research on whether the effect of individual characteristics on smoking behavior differs with neighborhood settings is still lacking.

China is the world’s largest producer and consumer of cigarettes [24]. It was estimated that 2 million Chinese smokers would die each year from tobacco-related diseases, such as lung, tracheal, and bronchial cancers by 2020 [25]. Hence, it is urgent to find an effective way to reduce the prevalence of tobacco use. In China, uneven economic reforms and decentralization have created inequalities among different social sectors, especially in the regional gap between rural urban and inland coastal areas [26,27]. Deprived communities mostly located in rural and inland areas may have difficulty effectively implementing smoking regulations due to environmental factors that facilitate smoking and inadequate health facilities, knowledge, and regional monitoring [28,29,30]. Previous studies found that tobacco control policies lowered smoking prevalence among females and in urban areas, whereas smoking prevalence in rural areas and among male heavy smokers (≥20 cigarettes daily) did not decrease [31,32]. It is urgent to find an effective way to reduce the intensity of tobacco use in China. Since smoking intensity is correlated to physical and mental health [33,34], this study explores the influences of cross-level predictors on smoking intensity.

On the basis of data from the China Health and Nutrition survey (CHNS) in 2015, this research aims to extend previous studies by investigating the relative and interaction effects of individual characteristics and community contextual factors on smoking behavior among Chinese adults by utilizing a cross-level model. Community deprivation, individual perceived stress, and socioeconomic status were analyzed to determine their possible associations with smoking intensity while controlling for age and marital status. The results of this study provide a new avenue through which to mitigate tobacco prevalence in China. 

## 2. Theoretical Review and Research Questions

### 2.1. Theoretical Review

“Health-related behavior is not just a matter of personal free choice and individual responsibility, but rather needs to be placed within a broader context that emphasizes structural constraints as well as choices” [35] (p. 42). To articulate correlations between the microscale of people and the macroscale of contextual settings, the cross-level perspective of health-related behavior was suggested by Duncan et al. in 1996 [5]. Thus, smoking behavior need to be explored through both macro-and micro-level geographical processes, including globalization, urbanization, increased area deprivation, and inequality that may originate personal stresses that are directly linked to smoking prevalence [6]. The social and economic development of a community shapes smoking behaviors through place-based practices and regulations [36]. On the one hand, less developed circumstances create chronic life difficulties that may trigger tobacco use through which to relieve stressful life events [37]. On the other, social and political measures showed correlation with smoking prevalence [38]. Previous research found a great likelihood of being a current smoker for individuals living in provinces with the highest rate of cigarette production compared to those with the smallest in China [39]. 

As an area develops rapidly, another mechanism leading to the convergence of smoking prevalence concerns individual characteristics such as gender, stress, and socio-economic status. As women work to earn money outside the home, they simultaneously receive double stress from workload and family chores [40], which may be eased with smoking. Prior studies found that smoking is a coping response to stressful neighborhood environments, especially for low SES women who resided in more affluent neighborhoods in Taiwan [22]. In addition, manual work and area deprivation resulted in higher smoking prevalence among women in affluent urban areas in South Korea [20]. 

While the prevalence of smoking is not extremely gendered in the Western context [41], the divergence among genders in smoking is a neglected topic in Asian society. In China, the low level of smoking among women is attributed to the social stigma against women smoking, whereas male smoking is accepted in all spheres of life from economic activity to leisure pastime [42]. Even though there are male smokers whose educational and occupational image are not appropriate for smoking tobacco, their smoking rates are similar to or even exceed those of the general population, due to intrinsic cultural factors [43,44]. On the basis of the theoretical review, in this study a cross-level model including variables of gender, stress, socio-economic status, and community deprivation was constructed to investigate smoking behavior among Chinese adults. 

### 2.2. Research Questions

Various studies conducted globally and in China verified individual factors and the environmental context as important determinants of smoking behavior. Some studies reported correlations between individual mental status (perceived stress and depression) and smoking behavior by focusing on specific vulnerable social groups [45,46]. For instance, one study found that rural–urban migrant workers with high perceived life stress showed a 45% excess odds ratio in terms of smoking [45]. Others found that socioeconomic status (educational attainment, occupation, income, and wealth) directly affect smoking behaviors [47,48,49]. These studies exclusively focused on individual variation, which neglects the potential importance of macrolevel attributes for smoking-related outcomes. 

Meanwhile, a number of studies revealed an association between the context of a geographical area and smoking behavior without accounting for the influence of individual-level variation on this effect [4,50,51]. Therefore, multilevel studies examining how the social context and individual-composition-related factors affect smoking behavior are warranted, although some studies in Asian societies have already been conducted [20,21,22,23]. In Japan, women in less residentially stable neighborhoods were more likely to smoke, whereas women in the most deprived neighborhoods were more likely to quit smoking [21]. Nonetheless, the combined influence of individual characteristics and environmental contexts on smoking behavior remains poorly understood due to insufficient case studies. To clarify the relative and interaction effects of multilevel factors on smoking behavior, the following research questions are proposed.

1. What is the relationship between area-level deprivation and smoking behavior after adjusting for within-community variation in individual characteristics?
2. Does the relationship between individual characteristics and smoking behavior differ with area-level deprivation?
3. Is the moderation effect between individual characteristics and area-level deprivation on smoking behavior gendered in the Chinese context?

## 3. Materials and Methods

### 3.1. Data Set and Variables

This study utilized the cross-sectional data collected from the China Health and Nutrition Survey (CHNS) in 2015, which was conducted by a multistage, random cluster process to survey individuals and households within 288 communities within twelve provinces in China. These twelve provinces contain approximately 45% of the Chinese population. To obtain the sample, counties inside the provinces were stratified by income (low, middle, and high), then a weighted sample of four counties were selected. Within these counties, neighborhoods (villages) were randomly chosen within 288 communities. Finally, a total of 7319 households was randomly selected from these neighborhoods (villages) and 20,914 household members were interviewed. After removing incomplete responses and inappropriate data, 10,815 samples of over 21-year-olds were selected for statistical analysis (for detailed information, please see https://www.cpc.unc.edu/projects/china/about/design/ accessed on 5 September 2020). 

Smoking behavior was measured with the question, “How many cigarettes do you smoke per day?”, with responses ranging from “none” to “60”. Individual-level SES was calculated from two indicators: educational attainment and employment status. Education was measured by asking respondents, “What is the highest level of education you have attained?” with responses ranging from “graduated from primary school” to “master’s degree or higher”. The six categories were recoded as “1 = college” and “0 = below college”. Employment status was measured by asking participants, “What is your primary occupation?” and “Why are you not working?”. Employment status was recoded from a number of options and grouped into three categories: employed in white collar work (managerial, professional, and clerical jobs), employed in blue collar work (manual, service, and farmer), and others (seeking work, retired, homemaker, student, unemployed, and disabled). 

The Perceived Stress Scale (PSS-10) was designed to measure an individual’s perception of stressors [52] and has been verified as measuring perceived stress in a large community-based general population in the Chinese context [53]. In the CHNS 2015 survey questionnaire, the related answers were recorded on a 5-point Likert scale ranging from “0 = never” to “4 = very often”. Positively framed questions (questions 6, 7, 9, and 10) were reverse scored (“4 = never” to “0 = very often”). The scores were summed, with higher scores indicating higher levels of perceived stress. The cutoffs for different levels of stress were selected in accordance with previous studies in China [54,55]. The levels of stress were coded as follows: “1 = high perceived stress (stress score ≥ 27)”, “2 = moderate perceived stress (stress score between 14 and 26)”, and “3 = low perceived stress (stress score ≤ 13)”. 

Given the multidimensional aspects of poverty, this study used a composite indicator of various variables to measure community deprivation. These variables include community-level education, health quality, social services, sanitation, and housing [56,57]. As area-level SES indicators, these five dimensions are equality important [58], were standardized using the z-score technique and were summed to provide an overall deprivation score for each community [59]. For the 288 community in the study area, the median score was 0.356 (standard deviation = 3.471). Area deprivation was then converted into three categories ranging from 1 (most deprived < −2.778) to 3 (least deprived > 2.905). Table 1 shows the descriptions for each compounded dimension. Age and marital status were included in the analysis as control variables. Age is a continuous variable, and marital status was recorded as “1 = never married”, “2 = married”, and “3 = other (divorced, widowed, separated)”. To better target both sexes in order to reduce cigarette intensity in China, statistical results were reported for each sex. The reasons that we analyzed the two sexes separately are as follows. First, the gap in tobacco use between the two sexes is significant. According to the global adult tobacco survey (GATS) in China in 2018, from a total of 307.6 million adults, 50.5% of men currently smoked tobacco compared with only 2.1% of women [34]. Second, previous studies reported that sex both moderated the effect of socioeconomic status on smoking behaviors, and interacted with area indicators to influence smoking prevalence [60,61,62]. 

### 3.2. Statistical Methods

In this study, an analysis was performed to examine the association between multilevel predictors and smoking behavior in China. Multilevel regression analysis was conducted to estimate the relative and interaction effect between individual characteristics (perceived stress, education, and employment status) and community deprivation on individual smoking intensity. The multilevel data structure considered in the present analysis comprised 10,815 individuals (level 1) nested within 288 communities (level 2). The current study tested four sets of multilevel regression models (random intercept models). These models can provide estimates of the relative and interaction compositional effects (individual) and contextual effects (community) on smoking intensity. To test these research questions, individual variables (individual-level fixed parameters) and community deprivation variables (community-level fixed parameters) were fitted. The intercept was assumed to be random at level 2 in all models. All statistical analyses were conducted using the statistical package SPSS 21, and linear mixed modeling (LMM) was used to estimate the multilevel regression model [63,64]. The following are detailed descriptions of these models: 

Model 0: This is a two-level null (empty) model of individuals nested within communities (level 2) with only a constant term in both the fixed and random parts. Variation in smoking behaviors is partitioned across individuals (within communities) and between communities. 

Model 1: Given that there is considerable variability between communities in the likelihood of forming smoking behaviors among different social groups, this model explains this variability. This model includes all individual-level predictors (perceived stress, educational attainment, and employment status) in the fixed part. The model assesses the effect of individual-level predictors on smoking behavior. 

Model 2: This model adds community-level predictors to assess whether this variable explains the variability in community intercepts. Thus, in addition to all the individual-level predictors, this model also includes community deprivation scores to verify the particular effect of community-level predictors on smoking behavior while controlling for individual-level predictors. 

Model 3: This model assesses whether variables measured at a higher level of the data hierarchy (community deprivation) enhance or diminish the correlations observed at a lower level of the hierarchy (individual characteristics and smoking behavior). Specifically, this model includes cross-level interactions to explore the moderating effect of community deprivation on the relationship between individual characteristics and smoking intensity. 

## 4. Results

Table 2 presents descriptive statistics for the outcome and predictor variables by sex. Over half of the male respondents currently smoked, while only 2% of the female respondents currently smoked. The average number of cigarettes smoked per day among males was over seven, while the number for females was less than one. Both sexes had similar mean values of perceived stress. The average age in the sample was approximately 51years old, and a large majority of respondents were married at the time. Approximately 13% of the sample had completed college. In terms of employment status, white collar workers made up the smallest proportion (13%), while those with a status of other (seeking work, retired, homemaker, students, unemployed, and disabled) made up the largest proportion (53.9%). Over one-fifth of the respondents of each sex resided in the most deprived areas. 

Table 3 and Table 4 provide the results of the multilevel regression analyses for both sexes. The null model with no predictors (model 0) revealed significant variation in smoking behaviors between communities. The variance component output indicates that the proportion of between-community variance in smoking behavior was 0.064 of the total variation for males (ICC = 6.313/91.807 + 6.313) and 0.039 for females (ICC = 0.217/5.434 + 0.217). The ICC suggests that approximately 6% and 4% of the total variability in smoking behavior for both sexes lay between communities. Thus, a multilevel model can be developed to explain the variability in the intercepts within and between communities. In addition, the results of the null model suggest that the development of a multilevel model is warranted because the intercepts vary significantly across communities (Wald Z = 6.426, p<0.001; Wald Z = 5.042, p<0.001). 

Model 1 estimates that male respondents (Table 3) who had moderate stress, had never married, and had a college degree were less likely to smoke than those in the comparison group. In addition, males who engaged in blue collar work were more likely to smoke (Wald Z = 5.717, p<0.001). Among female respondents (Table 4), those who had moderate stress and were married were less likely to smoke than those with other stress levels or who were unmarried, whereas females who had high stress were more likely to smoke more cigarettes than those with less stress (Wald Z = 5.033, p<0.001). In model 2 (Table 3 and Table 4), community deprivation exhibits statistically significant associations with smoking intensity among both sexes after adjusting for individual characteristics (Wald Z = 5.53, p<0.001;Wald Z=4.429, p<0.001). In other words, in communities with high deprivation levels, both males and females were more likely to smoke more cigarettes. 

In model 3, after including interactions between individual characteristics and community deprivation, only community deprivation significantly moderated the relationship between perceived stress and smoking intensity. Specifically, males (Table 3), who had moderate stress and resided in the most deprived communities were less likely to smoke than individuals who had the same level of stress but resided in the least deprived communities (Wald Z = 5.451, p<0.001). For females (Table 4), those who had high stress and resided in the most deprived communities were more likely to smoke more cigarettes than individuals who had the same level of stress but resided in the least deprived communities. Similarly, females who had moderate stress and resided in the most deprived communities were also less likely to smoke than individuals who had the same level of stress but resided in the least deprived communities (Wald Z = 4.241, p<0.001).

## 5. Discussion

Rapid economic development in the past three decades has enhanced purchasing power in China. As growth in income has been higher than growth in tobacco prices, the ability to pay for cigarettes in 2016 was almost 1.9 times that in 2001 [65]. Although China joined the World Health Organization (WHO) Framework Convention on Tobacco Control (FCTC) in 2006, Chinese smoking rates remain high [66]. Specifically, men aged 25–64 years, those who reside in rural areas and poorer western regions, and blue-collar workers were verified as groups with a high prevalence of smoking [34,66]. With growing independence and the marketing of tobacco towards women, female smoking is perceived as more acceptable in cities [67]. Given these tremendous changes in the social context, this study aims to investigate how the combination of community factors and individual characteristics contributes to smoking behavior in China. 

This study adds to the literature by demonstrating three major findings. First, neighborhood deprivation was associated with smoking intensity after adjusting for individual-level factors. This finding lends support to previous studies that showed a relationship between higher neighborhood poverty and increased smoking prevalence in Western societies [4,16]. In Western countries, neighborhood deprivation is strongly related to the outlet density of tobacco retailers. Previous studies found that outlets selling potentially health-damaging goods, such as tobacco, alcohol, and fast food increased linearly from the least to the most income deprived areas [13,68]. In China, the Tobacco Monopoly Bureau is responsible for tobacco enforcement, but no detailed provisions were documented in terms of inspections or sanctions [69]. Meanwhile, the Tobacco Monopoly Bureau issues government licenses for the sale of tobacco; however, any type of store or business can apply to sell tobacco [69]. Thus, in deprived rural areas where pressure from cigarette sharing and gifting customs permeates everyday life [28], it is difficult to control smoking prevalence in these pro-smoking and low-regulation circumstances. In addition, there is a lack of access to learning about the harmful side of smoking and second-hand smoking for rural residents compared with their urban counterparts [70]. Therefore, individuals who reside in deprived areas are more vulnerable to smoking practice.

Second, several cross-level interactions were also discovered in multilevel analysis. Community deprivation generally moderates the relationship between perceived stress and smoking behaviors. People of both sexes who lived in the most deprived communities and had moderate stress were less likely to smoke more cigarettes than people who lived in the least deprived communities. These results, on the one hand, may be attributed to the tobacco retail environment in affluent (the least deprived) urban areas in China. Previous studies found that the prevalence of tobacco advertisements and the density of tobacco retail outlets in urbanized neighborhoods were higher than those in less developed neighborhoods [71,72]. Thus, compared with rural deprived areas, individuals who reside in the least deprived urban areas may be exposed to an environment with higher levels of tobacco-related promotion, which, in turn, could stimulate their consumption. Meanwhile, people who have moderate stress may be less likely to depend on smoking to arouse a good mood compared to people with higher stress [14,15]. Hence, it is possible to explain why people with moderate stress who live in deprived communities were less likely to smoke than those who lived in affluent urban communities.

Third, females who lived in the most deprived communities and with higher stress were more likely to smoke more cigarettes than those with less stress in less deprived areas. This interaction effect demonstrates a situation in which women are doubly jeopardized, suggesting that females with high perceived stress who live in a deprived community intensify their cigarette smoking. Positive correlation between high stress and community development and smoking intensity for females may be attributed to stronger nicotine dependence among women who have high levels of perceived stress than those among comparable men [73]. In addition, smoking rates in deprived communities may be affected by increased levels of stress. Lacking access to resources, overcrowding, and social disorganization provoke a higher level of stress in deprived areas [74]. In line with this study, prior research conducted in Western societies found that higher neighborhood poverty was associated with increased smoking prevalence among Black US women [16].

The results of the current study suggest that individuals who live in deprived communities may be influenced by their context. Community-level interventions to decrease the prevalence of smoking among both sexes should be considered in China. On the basis of the results of this study, interventions targeting smoking control through individual characteristics may have short-term effects without sustainable community effects. Similarly, environmental changes may be insufficient to improve personal smoking behavior without supporting the cultivation of motivation. In practice, health-related behaviors are maximized when environmental settings support healthy choices and when individuals are motivated to make those choices [75]. At the community-level, substantial smoking regulations and anti-smoking campaigns should be implemented. On the individual-level, regular tobacco-cessation programs should be provided to local residents. Overall, multidimensional tobacco intervention strategies need to be implemented to advance the Sustainable Development Goals (SDGs) related to tobacco control in China, especially among females with higher stress who live in the most deprived communities.

The limitations of the study are as follows. First, the work was a cross-sectional study and based on self-reports in the 2015 survey. Longitudinal data should be considered in future research to better determine causality. In addition, smoking behavior may have changed, especially after the COVID-19 pandemic. Self-reported frequency was also a limitation because of memory inaccuracy. Therefore, future studies should take the pandemic period into account and a follow-up study is needed.

Second, regional-level GDP and individual-level income may correlate with smoking intensity in the Chinese context. Therefore, future studies should consider the effect of economic variables at both the regional and the individual-level when designing a multi-level model to investigate smoking behavior.

## 6. Conclusions

The aim of this study was to demonstrate the correlation among individual characteristics, community-level deprivation, and smoking behavior among Chinese adults using cross-level modeling. The results showed that being employed and having less than a college degree were positively associated with smoking intensity among males. Community-level deprivation was positively associated with smoking intensity after controlling for individual characteristics. Additionally, community-level deprivation differentially moderated the relationship between individual perceived stress and smoking intensity by sex. These findings suggest that community deprivation may influence smoking intensity directly and through interactions with individually perceived stress. On the basis of these findings, this study concludes that community-level influences should be addressed when developing multidimensional interventions and health policies to decrease tobacco use, especially for females with high perceived stress who live in deprived communities. Future research is needed to identify other contextual characteristics through which communities can influence smoking behavior in China.

## Figures and Tables

**Table 1:** Dimensions, indicators and descriptions of community deprivation score.

Dimensions	Descriptions	Range of Values
Education	Average education level among adults >21 years old	0.48–9.52
Health Quality	Number and type of the health facilities in or nearby (12 km) the community and number of pharmacies in the community	0–10
Social Services	Provision of preschool for children under 3 years old, availability of commercial medical insurance, free medical insurance, and insurance for women and children	0–10
Sanitation	Proportion of households with treated water and prevalence of households without excreta outside the home	0–10
Housing	Average number of days a week that electricity is an available to the community, percentage of community with indoor tap water, percentage of community with flush toilets, and percent of community that cooks with gas	0–10

Source: [58] (p. 5).

**Table 2:** Descriptive statistics for individual- and community-level variables used in models stratified by gender, restricted to ages over 21, CHNS, 2015.

Variables	Male (*n* = 5092)		Female (*n* = 5723)	
	Mean/%	SD	Mean/%	SD
Smoking behavior	7.34	9.902	0.3	2.373
Individual-level variables				
Perceived stress	15.51	4.783	15.78	4.829
Age	51.62	18.44	51.77	18.62
Marital status		0.343		0.384
Never married	6.7%		4%	
Married	88.2%		84.7%	
Others	5.1%		11.3%	
Education		0.354		0.333
College and over	14.7%		12.7%	
Below college	85.3%		87.3%	
Employment status		0.713		0.71
White collar	14.9%		12.7%	
Non-white collar	40%		25.5%	
Others	45.1%		61.8%	
Community-level variables				
Community deprivation		0.704		0.7
Most deprived	23.2%		22.6%	
Middle deprived	50.4%		50.7%	
Least deprived	26.4%		26.6%	

**Table 3:** Fixed and random part results for the multilevel regression models of male respondents, restricted to ages over 21, CHNS, 2015.

	Model 0	Model 1	Model 2	Model 3
Constant	7.312	7.527	6.398	5.679
Individual-level predictors				
Perceived stress				
High stress		−0.249	−0.212	1.206
Moderate stress		−0.652 * (−1.251, −0.053)	−0.689 * (−1.288, −0.089)	0.049
Age		−0.002	−0.002	−0.002
Marital status				
Never married		−2.105 ** (−3.684, −0.527)	−2.098 ** (−3.675 −0.52)	−2.105 *** (−3.683, −0.528)
Married		−0.289	−0.26	−0.265
Education (College = 1)		−2.853 *** (−3.74, −1.966)	−2.586 *** (−3.485, −1.68)	−2.718 *** (−3.611, −1.83)
Employment status				
White collar		1.871 *** (0.972, 2.771)	1.934 *** (1.035, 2.834)	1.873 *** (0.976, 2.771)
Non-white collar		2.221 *** (1.623, 2.819)	2.167 *** (1.569, 2.765)	2.102 *** (1.505, 2.699)
Community-level predictors				
Community deprivation				
Most deprived			1.821 *** (0.762, 2.881)	3.154 *** (1.526, 4.782)
Middle deprived			1.367 ** (0.481, 2.253)	1.833 ** (0.549, 3.119)
Cross-level interaction				
Most deprived × High stress				−6.125
Most deprived × Moderate stress				−1.801 * (−3.539, −0.063)
Middle deprived × High Stress				−0.259
Middle deprived × Moderate Stress				−0.713
Random parameters				
Between communities	6.313 ***	4.964 ***	4.624 ***	4.448 ***
Intraclass correlation (ICC)	0.064	0.052	0.049	0.048

Notes: * *p* < 0.05; ** *p* < 0.01; *** *p* < 0.001; 95% confidence interval was presented in parenthesis only for predictors that had significant effects on smoking behavior. Perceived stress (reference = low); Employment status (reference = other), Marital status (reference = others); Community deprivation (reference = least deprived). The interaction results between community deprivation and individual socioeconomic statuses are not shown in this table due to the insignificant effects on smoking intensity.

**Table 4:** Fixed and random part results for the multilevel regression models of female respondents, restricted to ages over 21, CHNS, 2015.

	Model 0	Model 1	Model 2	Model 3
Constant	0.306	0.728	0.629	0.679
Individual-level predictors				
Perceived stress				
High stress		0.939 ** (0.285, 1.592)	0.916 ** (0.263, 1.568)	−0.454
Moderate Stress		−0.237 *** (−0.376, −0.097)	−0.264 *** (−0.403, −0.125)	−0.33 ** (−0.585, −0.076)
Age		0.0009	0.0007	0.0007
Marital status				
Never married		−0.225	−0.237	−0.219
Married		−0.316 ** (−0.512, −0.12)	−0.333 *** (−0.529, −0.138)	−0.33 *** (−0.525, −0.136)
Education (College = 1)		−0.214	−0.162	−0.178
Employment status				
White collar		−0.003	0.029	0.006
Non-white collar		−0.018	−0.035	−0.043
Community-level predictors				
Community deprivation				
Most deprived			0.493 *** (0.265, 0.722)	0.908 *** (0.53, 1.285)
Middle deprived			0.047	−0.193
Cross-level interaction				
Most deprived × High stress				2.09 * (0.272, 3.909)
Most deprived × Moderate stress				−0.544 ** (−0.95, −0.134)
Middle deprived × High Stress				1.777 ** (0.249, 3.306)
Middle deprived × Moderate Stress				0.323
Random parameters				
Between communities	0.217 ***	0.215 ***	0.177 ***	0.16 ***
Intraclass correlation (ICC)	0.039	0.038	0.032	0.024

Notes: * *p* < 0.05; ** *p* < 0.01; *** *p* < 0.001; 95% confidence interval was presented in the parenthesis only for predictors that had significant effects on smoking behavior. Perceived stress (reference = low); Employment status (reference = other), Marital status (reference = others); Community deprivation (reference = least deprived). The interaction results between community deprivation and individual socioeconomic statuses are not shown in this table due to the insignificant effects on smoking intensity.

## Data Availability

After approval of use, publicly available datasets were analyzed in this study. https://www.cpc.unc.edu/projects/china/data/datasets/longitudinal/datasets accessed on 5 September 2020.
